# Assessment of physical activity and inactivity in multiple domains of daily life: a comparison between a computerized questionnaire and the SenseWear Armband complemented with an electronic diary

**DOI:** 10.1186/1479-5868-9-71

**Published:** 2012-06-12

**Authors:** Tineke Scheers, Renaat Philippaerts, Johan Lefevre

**Affiliations:** 1 Department of Kinesiology, KU Leuven, Tervuursevest 101, 3001, Leuven, Belgium; 2 Research Foundation, Flanders, Belgium; 3 Department of Movement and Sport Sciences, Ghent University, Watersportlaan 2, 9000, Ghent, Belgium

**Keywords:** Measurement, Validity, Self-report, Activity monitor, Activities of daily living, Sedentary behavior, Epidemiology

## Abstract

**Background:**

Although differences between paper-and-pencil questionnaires and accelerometers have been reported for overall physical activity and time spent in moderate and vigorous activity, few studies have looked at domain-specific behavior. This study compared estimates of domain-specific physical (in)activity obtained with the Flemish physical activity computerized questionnaire (FPACQ) with those obtained from a combination of the SenseWear Armband and an electronic diary. Furthermore, it was investigated whether the correspondence between the two methods varied with gender and age.

**Methods:**

Data were obtained from 442 Flemish adults (41.4±9.8 years). Physical activity was questioned with the FPACQ and measured for seven consecutive days using the SenseWear Armband together with an electronic activity diary (SWD). Analogous variables were calculated from the FPACQ and SWD. Mean differences and associations between FPACQ and SWD outcomes were examined with paired t-tests and Pearson correlations. The Bland-Altman method was used to assess the level of agreement between the two methods. Main effects and interaction of gender and age groups (20–34; 35–49; 50–64 years) on differences between FPACQ and SWD outcomes were analyzed using two-way ANOVAs.

**Results:**

All parameters of the FPACQ were significantly correlated with SWD assessments (r = 0.21 to 0.65). Reported activity was significantly different from SWD-obtained values for all parameters, except screen time. Physical activity level, total energy expenditure and time spent in vigorous activities were significantly higher (+0.14 MET, +25.09 METhours·week^-1^ and +1.66 hours·week^-1^, respectively), and moderate activities and sedentary behavior significantly lower (-5.20 and -25.01 hours·week^-1^, respectively) with the FPACQ compared to SWD. Time and energy expenditure of job activities and active transport were significantly higher, while household chores, motorized transport, eating and sleeping were significantly lower with the FPACQ. Time spent in sports was lower (-0.54 hours·week^-1^), but energy expenditure higher (+4.18 METhours·week^-1^) with the FPACQ. The correspondence between methods varied with gender and age, but results differed according to the intensity and domain of activity.

**Conclusions:**

Despite the moderate correlations, significant differences between the two methods were found. In general, physical activity was higher and sedentary behavior lower as calculated from the FPACQ compared to SWD.

## Introduction

The accurate assessment of physical activity (PA) and sedentary behavior in free-living conditions has always been a challenge in epidemiological research [1]. Continued efforts to improve assessment techniques are critical for systematic advancements of the field [2]. The accurate measurement of PA is important to clarify the strength and nature of the dose–response relation between PA and health, to identify current and changing activity levels within populations, to monitor adherence to activity guidelines and to determine the effectiveness of interventions designed to promote PA [3].

Self-report techniques remain the most widely used method to evaluate activity patterns at population level [4]. Although, over the past decade, activity monitors such as accelerometers are being used more frequently to objectively characterize PA behavior [2,5]. However, both methods have their limitations and when comparing subjective techniques with accelerometry, major discrepancies may emerge.

Significant errors in subjective measures of activity may occur due to several reasons. First, the accuracy of self-reports is often limited by the cognitive demands of recall, social desirability bias and misinterpretation of the questions [6]. Secondly, most questionnaires tend to focus on only one aspect of every day activity, such as work or leisure time. Few have been developed to assess PA and sedentary behavior in all major areas of daily life, namely leisure time, work, household chores and transport [7,8]. However, since leisure time PA accounts for only a small proportion of time and energy expenditure (EE) [9], it is unlikely that methods that are restricted to leisure time PA provide an accurate assessment of total daily EE. Third, people engage in a variety of lifestyle activities, which are intermittent and spread throughout the day [1]. While subjects can accurately recall structured bouts of vigorous activity, intended specifically for exercise, they are not as good at recalling routine or spontaneous, light to moderate activities [2,7].

Accelerometers provide objective data about the intensity, frequency and duration of PA, but they cannot assess the type of activity. Furthermore, accelerometers, typically placed on the hip, are unable to detect cycling, isolated arm movements, locomotion on a gradient or the added strain of lifting, carrying or pushing objects. As a result, PA is likely to be underestimated using accelerometry [10,11].

Thus, due to inaccurate recall, social desirability or omission of lifestyle activities in questionnaires and the inability of accelerometers to detect all activities equally well, both under- and over-reporting of activity in comparison with an accelerometer can occur [4,12].

To address these limitations, improvements in both subjective and objective measurements of PA are needed. Computerized questionnaires, like the Flemish physical activity computerized questionnaire (FPACQ) [13], have the advantage of a greater feeling of privacy and anonymity, compared to traditional written surveys. This results in a more honest reporting of sensitive information and a reduction of social desirability bias [14]. Nevertheless, the literature reveals few studies on the validity of computerized PA questionnaires. Furthermore, in contrast to most questionnaires, the FPACQ assesses PA and sedentary behavior in all domains of daily life.

Activity monitors like the SenseWear Armband, which combine accelerometry with physiological parameters, can improve the accuracy of measurement [15]. However, no single technique can capture all aspects of activity. Only self-reports can provide information on the type of activity [4,7]. Therefore, to allow for a more comprehensive investigation of activity patterns, we complemented the objective assessment through the SenseWear with an electronic activity diary. The combination of these two techniques made it possible to generate (in)activity variables in the same domains as derived from the FPACQ. Thus, in contrast to previous studies, which only used an overall PA score or time spent in moderate and vigorous activities, this study was able to compare subjective and objective measures of PA and sedentary behavior in all domains of daily life.

However, neither the FPACQ, nor the SenseWear is a golden standard for measuring PA. Therefore, we are limited to describing correspondence between both measures. Nevertheless, in the absence of a true criterion method, the SenseWear Armband combined with the electronic diary will be used as reference method.

The purpose of the present study was to compare estimates of domain-specific PA and sedentary behavior obtained with the FPACQ with those obtained from a combination of the SenseWear Armband and an electronic diary (SWD). Furthermore, it was investigated whether the correspondence between the two methods varied with gender and age.

## Materials and methods

### Subjects and study design

Participants were recruited from various companies and different work sectors (private companies, multinationals, education, research, social and welfare services, municipal services and industry) in Flanders, Belgium. Individuals volunteered to participate in the study and provided informed consent prior to participation. The study was approved by the Medical Ethics Committee of the KU Leuven. Subjects received an e-mail with a username and password and were asked to fill in the FPACQ at home via an internet platform. One to two weeks later health parameters were measured and PA monitoring devices were explained and provided to the subjects at their workplace. Subjects were asked to wear the devices 24 hours a day except during water-based activities, for the following 7 days. A total of 442 subjects (212 men and 230 women) between 22 and 64 years (mean age: 41.4 ± 9.8 years) participated in the study. To be included in analyses, subjects needed at least six valid monitoring days, including a Saturday and a Sunday [16]. A valid day was considered a day with at least 1368 min of data, which corresponds to 95% of a 24-hour period. Valid SenseWear and SWD data were available from 405 and 383 subjects respectively.

### Assessment of physical activity

#### The Flemish physical activity computerized questionnaire (FPACQ)

The FPACQ is a user-friendly computerized questionnaire that collects detailed information about patterns of PA and sedentary behavior in a usual week [13]. Three different versions of the questionnaire were developed to account for differences in lifestyle of population subgroups: students, employed/unemployed people and pensioners. The FPACQ for the employed/unemployed contains 59 to 103 closed-ended questions on demographic parameters (10 items), bouts of moderate and vigorous PA (3 to 6 items), total sedentary time (2 items), occupation (1 to 22 items), transport in leisure time (6 items), watching TV or playing computer games (2 items), household chores (3 items), eating (1 item), sleeping (1 item) and determinants of PA (29 items). Skip patterns are used to avoid superfluous questions. The web-based version of the questionnaire is available on http://www.FPACQ.be. At present, the FPACQ is only available in Dutch, but in the near future, French, English and Portuguese versions will be developed.

For the present study, 20 parameters were calculated from the FPACQ. Total sedentary time and bouts of moderate and vigorous PA were calculated from questions based on the short, self-administered version of the International Physical Activity Questionnaire (IPAQ) [17]. Job time represents the time spent on the main and additional occupation. Additionally, percentages of work time doing light, moderate and vigorous activities were questioned. Light activities were assigned a metabolic equivalent (MET) value of 2, moderate activities 3 and vigorous activities 4. Percentages were multiplied by the total job time and the assigned MET-values to calculate EE during work. Furthermore, subjects were asked to select a maximum of three of their most important sports out of a list of 200 specific sports. The weekly hours spent on these sports were summed to calculate time of sports participation. For each sport, the MET-value was determined using the Compendium of Ainsworth [18] and multiplied by the time spent on this sport. The sum of these multiplications resulted in EE during sports. Screen time is the sum of hours spent on watching TV or playing computer games during weekdays and weekend days. Time of household chores includes time spent on light, moderate and vigorous home and garden activities. These activities were assigned a MET-value of 2.5, 3.5 and 4.5, respectively, to calculate household EE. FPACQ queried about transport on foot and by bike for leisure and commuting to and from work. The results of these questions were summed and multiplied by 4 MET to estimate time and EE of active transport. Similarly, motorized transport was calculated from questions about transport with a car, train, tramcar, bus or motorcycle and a MET-value of 1.5. Time eating and time sleeping represent the hours spent eating and sleeping during a typical week. EE of these activities was estimated using a MET-value of 1.8 and 0.9, respectively. Finally, two general variables were calculated. Total EE represents the overall weekly EE and was calculated by summing the EE of all reported activities. Physical activity level (PAL, MET) was subsequently calculated by dividing total EE by 168 (=numbers of hours per week).

#### The SenseWear Pro 3 Armband

The SenseWear Pro 3 Armband (BodyMedia, Inc., Pittsburgh, PA, USA) is a multisensor body monitor, worn over the triceps muscle of the right arm. It enables continuous collection of various physiological and movement parameters through multiple sensors, including a two-axis accelerometer and sensors measuring heat flux, galvanic skin response, skin temperature and near body ambient temperature. Data from these sensors are combined with gender, age, body weight and height to estimate EE and PA intensity, using algorithms developed by the manufacturer (SenseWear professional software, version 6.1). Anthropometric measurements were obtained in the morning prior to the consecutive seven-day period by trained staff with subjects barefoot and in underwear. Body weight was measured to the nearest 0.1 kg using a digital scale (Seca, Hamburg, Germany). Height was measured to the nearest 0.1 cm using a portable anthropometer of Martin (GPM anthropological instruments, Zurich, Switzerland).

#### The electronic activity diary

The activity diary software program was developed at the Department of Kinesiology of the KU Leuven and stored in a Palm Z22 Personal Digital Assistant (Palm, Inc., Sunnyvale, CA, USA). The diary consisted of seven main categories: sleeping/resting, personal care, eating/drinking, job, leisure time, transport and household chores. The last three categories were divided into a number of subcategories, to allow subjects to specify their activity in more detail. Subjects were asked to register their activities in the electronic diary, each time a new activity was started, for the entire seven-day period. Dunton et al. [19] have shown that diary-reported activity levels from a similar Palm handheld computer corresponded well to objective indicators of activity. Furthermore, these diaries are thought to minimize errors associated with coding and recall of activity, because they enable subjects to add real-time information directly into an electronic medium [2]. First, information from the diary was used to substitute missing SenseWear data, due to removal of the Armband. Missing values for sleep were imputed with the mean MET-value and EE of observed sleep during all other nights. Missing data of personal care and swimming were substituted with a constant MET-value and associated EE according to the Compendium of Ainsworth (a MET-value of 2 and 6, respectively) [18]. Furthermore, information from the diary was synchronized with data of the SenseWear to obtain minute-by-minute data of physical (in)activity behavior. As a result, information was available for all four activity dimensions (intensity, duration, frequency and type).

Twenty parameters were calculated from SWD data, analogous to those from the FPACQ. Total EE (METhours·week^-1^) was calculated by summing minute-by-minute MET-values during the entire week, while PAL (MET) was calculated as the average of MET-values. Furthermore, time spent in different intensity levels (hours·week^-1^) was determined. Periods of at least 10 consecutive minutes with an intensity ≥3 but <6 and ≥6 MET were summed over the entire week to achieve bouts of moderate and vigorous activity, respectively. Total sedentary time was calculated from minutes with a MET-value ≤1.8 minus minutes spent sleeping. Additionally, time and EE of the different activities in the five domains of daily life were calculated. Time spent doing a particular activity (hours·week^-1^) was based on the information from the diary, while EE was calculated as weekly METhours during those specific activities, using SWD.

For subjects with only six valid monitoring days, a weekly average was estimated using the following formula: ((mean of parameter over 4 weekdays)*5) + parameter on Saturday + parameter on Sunday.

### Statistical analyses

Descriptive statistics (means and standard deviations) were calculated for all variables. Mean differences in activity variables between the FPACQ and SWD were examined with paired t-tests. Associations between FPACQ and SWD variables were analyzed using Pearson product–moment correlation coefficients. The Bland-Altman method was used to assess the level of agreement between the two measurement techniques. Variables used for the Bland-Altman analyses were total EE and EE during job activities, sports, household chores, active transport and motorized transport. Two-way analyses of variance with the difference scores of the FPACQ and SWD outcome (FPACQ outcome – SWD outcome) as dependent variable and gender, age group (20–34; 35–49; 50–64 years) and their interaction as independent variables were performed for each activity parameter. Tukey HSD tests were carried out for post hoc comparisons if significant differences were found. All statistical analyses were performed using the SAS statistical program, version 9.2 (SAS Institute, Cary, NC, USA). Statistical significance was set at *P* < 0.05.

## Results

Results of the Pearson correlations and paired t-tests between outcomes of the FPACQ and SWD are shown in Table 1. All parameters of the FPACQ were significantly and positively correlated with SWD values, with correlations varying from 0.21 to 0.65. Total EE calculated from the FPACQ was moderately correlated with the direct measure of the SenseWear (r = 0.44). Concerning time spent in different intensity levels, a moderate correlation was obtained for sedentary behavior (r = 0.54), but low correlations were found for moderate and vigorous PA (r = 0.27 and 0.21, respectively). Regarding time spent in different activity domains, correlations were moderate for job (r = 0.44 to 0.45), leisure time (r = 0.57 to 0.65), household chores (r = 0.39 to 0.46) and transport (r = 0.49 to 0.58) and generally low for eating and sleeping (r = 0.26 to 0.45).

**Table 1:** Comparison and relationship between FPACQ and SenseWear and/or electronic diary (SWD) parameters (Mean ± SD)

	**FPACQ**	**SWD**	***P***	Pearson correlation^*^
**Total physical activity**				
PAL (MET)	1.76 ± 0.23	1.62 ± 0.25	<0.001	0.44
Total EE (METhours.week^-1^)	296.49 ± 38.39	271.40 ± 41.09	<0.001	0.44
Moderate PA (hours.week^-1^)	2.47 ± 3.02	7.67 ± 7.14	<0.001	0.27
Vigorous PA (hours.week^-1^)	2.34 ± 2.68	0.68 ± 1.23	<0.001	0.21
Sedentary behavior (hours.week^-1^)	37.85 ± 16.65	62.86 ± 12.61	<0.001	0.54
**Job**				
Time (hours.week^-1^)	37.77 ± 10.12	33.08 ± 11.40	<0.001	0.44
EE (METhours.week^-1^)	89.12 ± 26.70	61.39 ± 25.63	<0.001	0.45
**Leisure time**				
Time sports (hours.week^-1^)	2.58 ± 3.44	3.12 ± 3.45	0.003	0.57
EE sports (METhours.week^-1^)	19.04 ± 29.51	14.86 ± 17.08	<0.001	0.65
Screen time (hours.week^-1^)	14.65 ± 7.85	14.70 ± 9.02	0.648	0.57
**Household chores**				
Time (hours.week^-1^)	10.20 ± 6.33	15.03 ± 9.36	<0.001	0.46
EE (METhours.week^-1^)	30.14 ± 19.71	36.78 ± 24.03	<0.001	0.39
**Transport**				
Time active transport (hours.week^-1^)	3.81 ± 3.87	2.45 ± 2.61	<0.001	0.49
EE active transport (METhours.week^-1^)	15.24 ± 15.49	8.21 ± 9.61	<0.001	0.51
Time motorized transport (hours.week^-1^)	6.33 ± 4.03	8.99 ± 4.31	<0.001	0.58
EE motorized transport (METhours.week^-1^)	9.50 ± 6.04	17.67 ± 9.03	<0.001	0.52
**Personal care**				
Time eating (hours.week^-1^)	5.62 ± 2.42	9.42 ± 3.62	<0.001	0.26
EE eating (METhours.week^-1^)	10.12 ± 4.35	15.86 ± 6.55	<0.001	0.26
Time sleeping (hours.week^-1^)	48.90 ± 7.01	56.19 ± 9.39	<0.001	0.45
EE sleeping (METhours.week^-1^)	44.01 ± 6.31	53.88 ± 7.96	<0.001	0.36

EE, energy expenditure; PA, physical activity.

Moderate PA: ≥3- < 6 MET; Vigorous PA: ≥6 MET; Sedentary behavior: ≤1.8 MET.

^*^ All correlation coefficients are significant at *P* <0.001.

Reported activity was significantly different from SWD-determined values for all parameters, except screen time (Table 1). PAL, total EE and time spent in vigorous activity were significantly higher (+0.14 MET, +25.09 METhours·week^-1^ and +1.66 hours·week^-1^, respectively), and time spent in moderate activity and sedentary behavior significantly lower (-5.20 and -25.01 hours·week^-1^, respectively) as calculated from the FPACQ compared to the SenseWear. Time and EE of job activities were significantly higher (+4.69 hours·week^-1^ and +27.73 METhours·week^-1^), while household chores (-4.83 hours·week^-1^ and -6.64 METhours·week^-1^), eating (-3.80 hours·week^-1^ and -5.74 METhours·week^-1^) and sleeping (-7.29 hours·week^-1^ and -9.87 METhours·week^-1^) were significantly lower with FPACQ than with SWD. Time spent in sports was lower (-0.54 hours·week^-1^), but EE higher (+4.18 METhours·week^-1^) with the FPACQ. With regard to transport, reported time and EE of active transport were higher (+1.36 hours·week^-1^ and +7.03 METhours·week^-1^), while those of motorized transport were lower (-2.66 hours·week^-1^ and -8.17 METhours·week^-1^), compared to SWD assessments.

**Figure 1:**
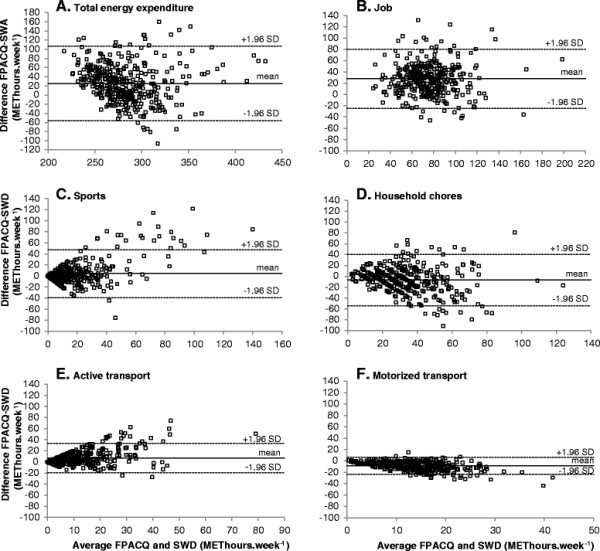
Bland-Altman plots for total energy expenditure (A) and energy expenditure during job activities (B), sports (C), household chores (D), active transport (E) and motorized transport (F), as assessed with the FPACQ on the one hand and the SenseWear Armband in combination with an electronic activity diary (SWD) on the other.

The Bland-Altman plots showed that FPACQ resulted in higher total EE for most subjects and throughout the range of values (Figure 1). Although the mean difference was fairly small (+25.26 METhours·week^-1^ or 9% of the average of FPACQ and SenseWear outcomes), 95% limits of agreement were wide, ranging from -56.15 to 106.67 METhours·week^-1^. EE during job activities was generally higher, while motorized transport was thoroughly lower with the FPACQ. Mean differences and 95% limits of agreement between FPACQ and SWD-determined EE were relatively wide for the various activity domains. No systematic bias was observed, except for EE during sports, where the over-reporting by the FPACQ increased with increasing EE.

Results of the two-way ANOVA to assess main effects and interaction of gender and age groups on differences between FPACQ and SWD outcomes are presented in Table 2. Time spent in vigorous activities, EE of sports and time and EE of active transport were significantly more over-reported in men, as compared to women. Furthermore, men underreported moderate PA and EE during sleep more, whereas women underreported total sedentary time and household chores to a greater extent. Screen time was underreported by men and slightly over-reported by women. Twenty to 34 year olds had smaller differences between FPACQ and SWD for PAL and total EE, but greater differences for time spent at moderate activities and EE during sleep than 35–49 and 50–64 year olds. Time spent in vigorous activities was more over-reported by young compared to middle-aged adults. Additionally, time during household chores was more underreported, and time and EE during active transport less over-reported in the second age group than in the oldest age group.

**Table 2:** Two-way ANOVA for differences between FPACQ and the combination of the SenseWear Armband and electronic diary (FPACQ - SWD outcome) with gender and age

	**Main effect of gender**		**Main effect of age**		
	**men (n = 196)**	**women (n = 209)**	**20-34 years (n = 104)**	**35-49 years (n = 204)**	**50-64 years (n = 97)**
**Total physical activity**					
PAL (MET)	0.16 ± 0.27	0.13 ± 0.23	0.08 ± 0.25^b,c^	0.15 ± 2.26	0.21 ± 0.21
Total EE (METhours.week^-1^)	27.64 ± 44.48	22.89 ± 38.35	13.98 ± 41.83^b,c^	26.17 ± 43.19	35.34 ± 34.59
Moderate PA (hours.week^-1^)	-6.91 ± 7.63^a^	-3.61 ± 5.87	-7.08 ± 7.68^b,c^	-4.61 ± 6.72	-4.43 ± 6.34
Vigorous PA (hours.week^-1^)	1.93 ± 2.98^a^	1.26 ± 2.17	2.03 ± 2.93^b^	1.28 ± 2.48	1.76 ± 2.45
Sedentary behavior (hours.week^-1^)	-23.00 ± 14.08^a^	-26.92 ± 14.79	-23.45 ± 14.60	-25.52 ± 14.53	-25.64 ± 14.61
**Job**					
Time (hours.week^-1^)	5.08 ± 10.48	4.41 ± 12.41	5.53 ± 13.38	4.52 ± 11.29	4.32 ± 9.77
EE (METhours.week^-1^)	30.71 ± 25.23	24.88 ± 28.02	23.66 ± 29.68	30.45 ± 26.32	26.25 ± 24.15
**Leisure time**					
Time sport (hours.week^-1^)	-0.42 ± 3.85	-0.56 ± 2.46	-0.42 ± 3.05	-0.50 ± 3.02	-0.55 ± 3.75
EE sport (METhours.week^-1^)	8.59 ± 28.41^a^	0.48 ± 12.90	6.50 ± 23.38	4.28 ± 21.82	2.26 ± 21.38
Screen time (hours.week^-1^)	-1.15 ± 7.82^a^	0.71 ± 7.82	-0.56 ± 8.27	0.60 ± 7.79	-1.49 ± 7.46
**Household chores**					
Time (hours.week^-1^)	-3.38 ± 8.54^a^	-6.20 ± 8.33	-4.26 ± 7.95	-5.78 ± 8.80^d^	-3.44 ± 8.40
EE (METhours.week^-1^)	-4.72 ± 24.14^a^	-8.63 ± 24.08	-7.03 ± 22.31	-8.75 ± 23.95	-2.12 ± 26.07
**Transport**					
Time active transport (hours.week^-1^)	1.66 ± 3.90^a^	1.01 ± 2.94	1.27 ± 3.66	1.01 ± 3.30^d^	2.07 ± 3.45
EE active transport (METhours.week^-1^)	8.06 ± 15.17^a^	5.78 ± 11.49	6.73 ± 14.14	5.68 ± 12.89^d^	9.62 ± 13.48
Time passive transport (hours.week^-1^)	-3.00 ± 3.84	-2.84 ± 3.70	-2.79 ± 4.06	-2.92 ± 3.75	-3.06 ± 3.51
EE passive transport (METhours.week^-1^)	-8.98 ± 9.34	-8.19 ± 7.19	-9.56 ± 8.83	-8.42 ± 7.58	-7.84 ± 6.87
**Personal care**					
Time eating (hours.week^-1^)	-3.69 ± 3.82	-3.99 ± 3.77	-3.23 ± 3.44	-3.90 ± 3.80	-4.40 ± 4.07
EE eating (METhours.week^-1^)	-6.11 ± 6.84	-5.57 ± 6.91	-5.57 ± 6.59	-5.73 ± 6.73	-6.33 ± 7.50
Time sleeping (hours.week^-1^)	-7.20 ± 7.29	-7.50 ± 7.10	-8.09 ± 7.78	-6.83 ± 6.98	-7.71 ± 6.94
EE sleeping (METhours.week^-1^)	-10.87 ± 7.83^a^	-9.06 ± 8.61	-13.67 ± 9.40^b, c^	-9.40 ± 7.39	-7.06 ± 7.43

Data are presented as means ± standard deviation; EE, energy expenditure; PA, physical activity; Moderate PA: ≥ 3-< 6 MET; Vigorous PA; ≥6 MET; Sedentary behavior: ≤ 1.8 MET.

^a^ Significant difference between men and women, *P* <0.05.

^b^ Significant difference between age groups 20–34 and 35–49 years, *P* <0.05.

^c^ Significant difference between age groups 20–34 and 50–64 years, *P* <0.05.

^d^ Significant difference between age groups 35–49 and 50–64 years, *P* <0.05.

No interaction effects were observed.

## Discussion

This study compared estimates of domain-specific PA and sedentary behavior obtained with the FPACQ with those obtained from SWD. Furthermore, it was examined whether the correspondence between the two methods varied with gender and age. All parameters of the FPACQ were significantly and positively correlated with SWD-outcomes. Nevertheless, significant differences between both methods were found. In general, PA was higher and sedentary behavior lower with the FPACQ compared to SWD. These results are similar to those of several other studies, which showed that, when compared to objective data obtained from accelerometers, questionnaires have acceptable validity, but generally overestimate PA [13,20,21]. However, previous studies are mostly limited to overall PA or time spent at moderate and vigorous activity, whereas the current study highlights the importance of examining domain-specific activity when investigating agreement between measurement techniques.

Correlations between the two methods varied between 0.21 and 0.65 and are similar to what is typically reported for PA questionnaires evaluated in adults [4,17,22]. An important contribution of this study is the comparison between subjective and objective measures of physical (in)activity in different domains of daily life. Correlations were moderate for job, leisure time, household chores and transport, but low for eating and sleeping. To our knowledge, only two studies divided accelerometer output into different domains according to the information obtained from an activity log, similar to what was done in the current study. Measures of occupational activity from Tecumseh and Baecke questionnaires were significantly correlated with Tracmor output during work (r = 0.26 to 0.50), but low or no correlations were found for indices of active leisure time. However, active leisure time included a wide range of activities, such as sports, household and garden activities [23]. Matton et al. [13] showed comparable correlations for active transport (0.49-0.55), but higher correlations for sports (0.47-0.77), TV viewing (0.69-0.83), occupation (0.78-0.88) and eating and sleeping (0.53-0.69). There were, however, subtle differences in the calculation of the FPACQ parameters.

Despite of the significant correlations, PAL and total EE were significantly higher and sedentary time significantly lower with the FPACQ as compared to the SenseWear. These results are consistent with findings from several previous studies [4,13,21]. However, it is unclear whether the differences between the two methods are due to errors in the FPACQ or to inherent limitations of the SenseWear. It has been shown that the SenseWear underestimates total EE by 4% compared with doubly labeled water [24,25]. This could partly explain the observed difference in total EE between FPACQ and SenseWear (9% of the average of FPACQ and SenseWear outcomes).

With regard to intensity of activity, reported duration of vigorous PA was higher, whereas moderate PA was lower than directly measured by the SenseWear. These complex patterns have been seen in several previous studies. It has been reported that people overestimate the amount of vigorous activity, while underestimating time spent in light and moderate activities [26], though some studies also found an over-reporting of moderate activities [21,27]. The FPACQ questions of time spent in moderate and vigorous PA inquire about overall activity in multiple domains of daily life. These questions are cognitively challenging, because several activities need to be taken into account and summed over the day [28]. Most subjects, asked about PA behavior, seem to think about vigorous or organized activities and not about routine activities like household chores or walking [29]. This underlines the importance of examining domain-specific activity when investigating agreement between measurement techniques. In the current study, time and EE of job activities and active transport were significantly higher and household chores, passive transport, eating and sleeping significantly lower with the FPACQ as compared to SWD. Furthermore, the FPACQ resulted in lower values for duration, but higher values for EE of sports. Few studies have compared self-reported activity in different domains with similar measures obtained from activity monitors. Matton et al. [13] showed that duration of eating and sleeping and watching TV in women were significantly lower and time and EE of sport, time of active transport and EE during occupation significantly higher when calculated from the FPACQ as compared to an accelerometer plus log. Reported duration of active leisure time was higher in men and slightly, but not significantly, lower in women. However, active leisure time included sports participation, active transport and house and garden activities. This could possibly point to an underreporting of household activities in women, analogous to the current study.

The correspondence between FPACQ and SWD varied with gender and age. However, no clear pattern was observed. Trends differed according to the specific intensity and domain of activity. Men over-reported more intense activity significantly more than women, whereas women underreported total sedentary time and household chores to a greater extent. Young adults had smaller differences between FPACQ and SWD for PAL and total EE, but greater differences for time spent at moderate activities than middle-aged and older subjects. Additionally, vigorous activities were more over-reported by young compared to middle-aged adults. The evidence on the role of gender in the agreement between self-report and direct measures of PA has been mixed, with some studies demonstrating better agreement in men [4,30], while others have reported better agreement in women [21,31]. Calabro et al. [32] found that for men, the 24-hour recall estimate of total EE was slightly higher than the SenseWear, whereas for women, it was slightly lower. Only a few studies investigated the impact of age in the accuracy of self-reports. It has been reported that PA questionnaires are especially challenging in older adults because of cognitive processes [33]. Furthermore, a substantial component of their PA, namely activities of daily living, is not captured by most self-report instruments [34]. A review of Ferrari et al. [30] showed that the validity of questionnaires varied with age, with lower coefficients observed for subjects older than 50 years. However, results could differ depending on the questionnaire used [21].

Bland-Altman analyses revealed a relatively small mean difference between FPACQ and SenseWear for total EE. However, 95% limits of agreement were large, suggesting that there are large individual differences in estimates from both methods. Most of the previous studies have reported agreement at the group level, but not at the individual level [20,35]. Calabro et al. [32] found a relatively small (38.5 kcal·day^-1^), not significant, difference between the 24-hour PA recall and SenseWear for group-level EE. However, differences in individual estimates ranged from -663 to 946 kcal·day^-1^. In the current study, no systematic bias was observed for total EE. Yet, for sports, a trend towards increased over-reporting by the FPACQ with higher values of EE was found. Other studies also indicated an increased difference with increasing PA. Good agreement existed between IPAQ and ActiGraph up to 1000 min of PA per week. However, as activity levels increased over 1000 min, the IPAQ tended to overestimate total PA [21]. Bland-Altman plots for the 24-hour recall versus the IDEEA and SenseWear illustrated a tendency of the 24-hour recall to underreport total EE in the least active and over-report in the most active subjects [32].

Several reasons could explain the disagreement between both measurement methods. Social desirability may at least partially explain the over-reporting of PA and underreporting of sedentary pursuits [6]. It has been shown that, over a seven-day period, social desirability bias is associated with over-reporting of PA by approximately 4–11 min·day^-1^[36].

A higher perceived intensity than objectively measured may also lead to differences [37,38]. Some questionnaires, including the FPACQ, ask about activities where physiological parameters like increased sweating, heart rate or breathlessness mark the intensity [1]. However, the perception of intensity depends on the age, gender and fitness of the person as well as on duration of activity [1,2]. Moderate activities could be perceived as vigorous, which may explain the over-reporting of vigorous and underreporting of moderate PA. Likewise, subjects could have overestimated the intensity of their occupational activities, resulting in higher EE in the FPACQ.

A third explanation might be the problems associated with recalling light to moderate activities of daily living. It has been shown that it is difficult to achieve accurate measures of light to moderate PA using self-reports, probably due to their unstructured and intermittent nature [2]. Aadahl et al. [39] have reported that subjects knew quite accurately how much time they slept, worked or watched TV, and how much time they spent on vigorous activities such as sports or heavy gardening. But, the duration of light activities at home was very difficult to remember. This could explain why particularly women underreported the duration of household chores. It is possible that women performed lighter activities, whereas men performed heavier gardening. Additionally, it may be that women accumulated intermittent household chores over the course of the day, whereas chores of men were more structured, making them easier to recall.

Another source of variability may be the result of algorithms used to convert activity data into EE [10,11,31]. The SenseWear estimates EE based on physiological and movement parameters, whereas the FPACQ relies on MET-values from a published compendium [18]. Reported activities were converted into an estimate of EE by assigning each activity a specific MET-value. Thus, a single estimate of the energy cost of a certain activity was used for all subjects. This does not allow for individual differences in EE [1,2,18]. However, evidence suggests that there is considerable inter- and intra-individual variability in the energy cost of activities, depending on the person’s sex, age, body mass, movement efficiency and environmental conditions in which the activity is performed [6,40]. It is remarkable that EE of sports was highly over-reported for men, but not for women. This could point to a potential overestimation of MET-values of certain sports, perhaps those with a higher intensity or those mainly practiced by men. However, it is also known that the SenseWear underestimates EE during very vigorous activities [41,42].

It is important to recognize that the disagreement is a result of limitations in both methods. The reported disagreement in literature may be related to limitations in the use of accelerometers. Part of the overestimation of PA in self-reports may be explained by activities that are not detected with accelerometers [37]. In addition, the wear-time of accelerometers varies between studies and is generally low, for example minimum 10 hours per day [20,34]. However, adults could be awake for up to 16 hours. Thus, during some of the time that the subjects were awake, activities were not registered. It is likely that this produced some bias in the data [12,43]. The SenseWear can address some of these limitations. By combining accelerometry with physiological sensors, it can detect the increased EE associated with cycling, upper body movement, carrying loads and walking on an incline [15]. Moreover, in this study, the wear time was standardized to 24 hours a day. However, the SenseWear is not without limitations. Similar to other activity monitors, it is known to overestimate EE of moderate activities and underestimate (very) vigorous and total EE [25,41,42]. Johannsen et al. [24] have noted that the SenseWear underestimated PA EE by 12.5% compared to estimates derived from doubly labeled water. This may have contributed to the observed differences between FPACQ and SWD for total EE and the EE of sports and active transport. Furthermore, the Armband cannot be worn during water-based activities. However, in this study, a constant MET-value was imputed to account for swimming and showering or bathing. Because of these limitations, under- or overestimation by the FPACQ can neither be confirmed nor refused and real activity levels probably lie between the subjective and objective assessments.

Some results might reflect limitations in the use of the diary. First, participants may forget to record short-during activities, such as active transport, leading to an underreporting of these activities in the diary. Second, contrary to what was expected, screen time was not different between methods. Yet, the pattern is complex, as men underreported and women slightly over-reported screen time. This could be due to the following difference. In the diary, subjects were forced to choose between activities, when several activities were performed simultaneously, whereas in the FPACQ, both activities could be reported. For example, when eating a meal in front of TV, subjects could have inserted eating into the diary, whereas they also counted this period of TV-viewing when answering the screen time question in the FPACQ. Also surprisingly, time spent on sports was lower in the FPACQ, as compared to the diary. Indicated hours of sports participation in the diary might include time devoted to changing, refreshment and socializing [6]. Furthermore, subjects knew they participated in a PA study and were monitored for their activity. Thus, because of a possible Hawthorne effect, participants could have performed more sports than usual, resulting in higher values in the diary. This points to a potential restriction of the study. The FPACQ assessed activity during a usual week, where SWD measured last week activity. This could, at least in part, explain the difference in job time between both methods. Subjects could have been monitored during a week with some vacation days or less work time than usual. However, the interpretation of a usual week is difficult and participants sometimes recall the last 7 days as a usual week [17,28].

Some other limitations should be considered when evaluating the results of this study. Participants volunteered to take part in the study. This may have led to a selection bias as most participants were highly-educated and had white-collar functions. Accordingly, the generalizability of these findings to the general working population may be restricted. Though, a previous study showed that agreement between self-reported and accelerometer-obtained PA did not differ between educational levels [20].

The current study investigated whether the correspondence between recalled and direct measures of PA varied with gender and age. However, trends in agreement may be influenced by several other characteristics, including BMI and cardiovascular fitness [27,31,37]. Additional research is needed to identify whether, and to what extent, these factors are associated with reporting bias.

A major strength of this study is the combination of the SenseWear Armband, a valid activity monitor [24,25], with the electronic diary. Each minute of SenseWear data was linked to the diary reported type of activity. In this way, activity variables from the questionnaire could be compared with an objective measure generated in the same dimension, thereby moving beyond examinations of overall PA or time spent at moderate and vigorous intensity. In addition, compared to previous studies examining agreement between measurement techniques [8], this study included a relatively large sample of men and women of diverse ages. Furthermore, the compliance for wearing the SenseWear and completing the diary was very high and only subjects with at least six days with a minimum of 22 hours and 48 min (95% of 24 hours) of data were included in the analyses.

The current results show that great care must be taken when interpreting self-reported and objectively measured PA. Clearly, the two assessment techniques are not interchangeable. Both instruments capture different aspects of a complex behavior. Activity monitors like the SenseWear, measure motion or movement, while questionnaires provide a behavioral description of activity patterns. As shown previously, subjective and objective methods are independently associated with health parameters, and in that way, self-reports should be used as an addition to objective indicators of movement [44]. Furthermore, it is important to recognize that the current recommendation to accumulate 30 min of PA on most days, is based on associations between self-reported PA and health outcomes. The magnitude of these associations may be severely attenuated by measurement error [30] and less than 30 min of PA as measured by an accelerometer, may provide significant health benefits [5]. Thus, the benefits of PA may even be greater than what is typically reported.

## Conclusions

In conclusion, our results show a moderate correspondence between FPACQ and SWD. Despite the moderate correlations, significant differences between both methods were found. In general, PA was higher and sedentary behavior lower as calculated from the FPACQ, compared to SWD. Furthermore, correspondence varied with gender and age. Though, no clear patterns emerged. Results differed according to the specific intensity and domain of activity. The appendix can be seen in Table 3

## Appendix

 The appendix can be seen in Table 3.

**Table 3:** Physical (in)activity parameters calculated from the FPACQ and the combination of the SenseWear Armband and electronic diary (SWD)

**Parameter**	**Questions and responses in the FPACQ**	**Calculation of FPACQ parameters**	**Calculation of SWD parameters**
Sedentary behavior (hours.week^-1^)	How much time do you usually spend sitting on a weekday/weekend day? This may include time spent sitting at a desk, visiting friends, reading, studying or watching television.	(Hours spent sitting on a weekday * 5) + (hours spent sitting on a weekend day * 2)	Sum of all minutes with a MET-value ≤1.8 MET minus minutes spent sleeping
	→ Response: <30 min.day^-1^ to ≥10 hours.day^-1^		
Moderate physical activity (hours.week^-1^)	1) During a usual week, on how many days do you do moderate physical activities? Moderate activities refer to activities that take moderate physical effort and make you breathe somewhat harder than normal, like carrying light loads, bicycling at a regular pace, or doubles tennis. Do not include walking. Think only about those activities that you did for at least 10 min at a time.	Number of days with moderate activities * hours per day spent on moderate activities	Sum of all bouts of at least 10 min with a MET-value ≥3 but <6
	→ Response: 0 to 7 days.week^-1^		
	2) How much time do you usually spend doing moderate physical activities on one of those days?		
	→ Response: 10 to ≥120 min.day^-1^		
Vigorous physical activity (hours.week^-1^)	1) During a usual week, on how many days do you do vigorous physical activities? Vigorous activities refer to activities that take hard physical effort and make you breathe much harder than normal, like heavy lifting, digging, aerobics or fast bicycling. Think only about those activities that you did for at least 10 min at a time.	Number of days with vigorous activities * hours per day spent on vigorous activities	Sum of all bouts of at least 10 min with a MET-value ≥6
	→ Response: 0 to 7 days.week^-1^		
	2) How much time do you usually spend doing vigorous physical activities on one of those days?		
	→ Response: 10 to ≥120 min.day^-1^		
Total EE (METhours.week^-1^)		1) EE of remaining inactive leisure time= (168 - time job - time sports - time household chores - time active transport - time motorized transport - time eating - time sleeping) * 1.5 MET	Sum of min-by-min MET-values over the entire week
		2) EE job + EE sports + EE household chores + EE active transport + EE motorized transport + EE eating + EE sleeping + EE remaining inactive leisure time	
PAL (MET)		Total EE/168	Average of min-by-min MET- values over the entire week
Time job (hours.week^-1^)	How many hours a week do you usually spend doing your main/additional occupation?	Hours per week spent on main occupation + hours per week spent on additional occupation	Sum of all minutes reported as ‘job’ in the electronic diary
	→ Response: 0-5 to >60 hours.week^-1^		
EE job (METhours.week^-1^)	During a usual week at your main/additional occupation, what percentage of the time do you engage in 1) light activities (sitting, standing without lifting or carrying weights,…), 2) moderate activities (lifting or carrying weights, walking continuously,…), 3) vigorous activities (lifting or carrying moderate to heavy weights, construction worker,…)	(Hours spent on main occupation * % light * 2 MET) + (hours spent on main occupation * % moderate * 3 MET) + (hours spent on main occupation * % vigorous * 4 MET) + (hours spent on additional occupation * % light * 2 MET) + (hours spent on additional occupation * % moderate * 3 MET) + (hours spent on additional occupation * % vigorous * 4 MET)	Sum of min-by-min MET-values where the indicated activity was ‘job’
	→ Response: 0 to 100% for each of the three intensity levels		
Time sports (hours.week^-1^)	1) What is your most important sport?	1) Conversion of the reported duration of the first, second and third sport into average hours per week over one year	Sum of all minutes reported as ‘sports’ in the electronic diary (All separately indicated sports were combined)
	→ Response: list of 200 different sports		
	2) How frequently do you perform your most important sport?	2) Hours per week spent on first sport + hours per week spent on second sport + hours per week spent on third sport	
	→ Response: 1 week.year^-1^ to >7 times per week		
	3) How much time do you spend on your most important sport?		
	→ Response: <7 hours during 1 week.year^-1^ to >20 hours.week^-1^		
	4) How many months a year do you practice your most important sport?		
	→ Response: 1 to 12 months per year		
	Same four questions for the second and third most important sport		
EE sports (METhours.week^-1^)		(Hours per week spent on first sport * MET-value first sport) + (hours per week spent on second sport * MET-value second sport) + (hours per week spent on third sport * MET-value third sport)	Sum of min-by-min MET-values where the indicated activity was ‘sports’
Screen time (hours.week^-1^)	How many hours do you usually spend watching TV/video or playing computer games on a weekday/weekend day?	(Hours per day spent on a weekday * 5) + (hours per day spent on a weekend day * 2)	Sum of all minutes reported as ‘watching TV/movies’ and ‘playing or working on the computer for leisure’ in the electronic diary
	→ Response: 0 to ≥6 hours.day^-1^		
Time household chores (hours.week^-1^)	How many hours a week do you usually spend doing home or garden activities of 1) light intensity (cooking, ironing, watering flowers,…), 2) moderate intensity (vacuuming, mowing lawn,…), 3) vigorous intensity (scrubbing, digging,…)	Hours per week spent on light household chores + hours per week spent on moderate household chores + hours per week spent on vigorous household chores	Sum of all minutes reported as ‘in-house activities’ and ‘garden activities’ in the electronic diary
	→ Response: 0 to >14 hours.week^-1^		
EE household chores (METhours.week^-1^)		(Hours per week spent on light household chores * 2.5 MET) + (hours per week spent on moderate chores * 3.5 MET) + (hours per week spent on vigorous chores * 4.5 MET)	Sum of min-by-min MET-values where the indicated activity was ‘in-house activities’ or ‘garden activities’
Time active transport (hours.week^-1^)	1) How many days a week do you usually walk to and from your main/additional occupation?	(Number of days * hours per day spent on walking to and from main occupation) + (number of days * hours per day spent on walking to and from additional occupation) + (number of days * hours per day spent on cycling to and from main occupation) + (number of days * hours per day spent on cycling to and from additional occupation) + (hours per weekday spent on transportation on foot in leisure time * 5) + (hours per weekday spent on transportation by bike in leisure time * 5) + (hours per weekend day spent on transportation on foot in leisure time * 2) + (hours per weekend day spent on transportation by bike in leisure time * 2)	Sum of all minutes reported as ‘transportation on foot’ and ‘transportation by bike’ in the electronic diary
	→ Response: 0 to 7 days.wk^-1^		
	2) How many minutes do you usually walk to and from your main/ additional occupation on such days?		
	→ Response: 0-10 to >120 min.day^-1^		
	3) In leisure time, how many minutes do you usually spend on transportation on foot, on a weekday/weekend day?		
	→ Response: no transportation in this way to >120 min.day^-1^		
	Same three questions for transportation by bike		
EE active transport (METhours.week-^1^)		Time active transport *4 MET	Sum of min-by-min MET- values where the indicated activity was ‘transportation on foot’ or ‘transportation by bike’
Time motorized transport (hours.week-^1^)	1) How many days a week do you usually make use of a car, train, tramcar, bus or motorcycle for commuting to and from your main/ additional occupation?	(Number of days * hours per day spent on motorized transport to and from main occupation) + (number of days * hours per day spent on motorized transport to and from additional occupation) + (hours per weekday spent on motorized transport in leisure time * 5) + (hours per weekend day spent on motorized transport in leisure time *2)	Sum of all minutes reported as ‘motorized transport’ in the electronic diary
	→ Response: 0 to 7 days.week-^1^		
	2) How many minutes do you usually spend on transportation by car, train, tramcar, bus or motorcycle to and from your main/additional occupation on such days?		
	→ Response: 0-10 to >120 min.day-^1^		
	3) In leisure time, how many minutes do you usually spend on transportation by car, train, tramcar, bus or motorcycle on a weekday/ weekend day?		
	→ Response: no transportation in this way to >120 min.day-^1^		
EE motorized transport (METhours.week-^1^)		Time motorized transport * 1.5 MET	Sum of min-by-min MET- values where the indicated activity was ‘motorized transport’
Time eating (hours.week-^1^)	How many minutes a day to you usually spend eating your daily meals?	Hours per day spent eating *7	Sum of all minutes reported as ‘eating/drinking’ in the electronic diary
	→ Response: 0-10 to >120 min.day-^1^		
EE eating (METhours.week-^1^)		Time eating * 1.8 MET	Sum of min-by-min MET- values where the indicated activity was ‘eating/drinking’
Time sleeping (hours.week-^1^)	How many hours a night do you usually spend sleeping?	Hours per night spent sleeping * 7	Sum of all minutes reported as ‘sleeping/resting’ in the electronic diary
	→ Response: <5 to >12 hours.night-^1^		
EE sleeping (METhours.week-1)		Time sleeping * 0.9 MET	Sum of min-by-min MET- values where the indicated activity was ‘sleeping’

FPACQ, Flemish physical activity computerized questionnaire; EE: energy expenditure.

## Competing interests

The authors declare that they have no competing interests.

## Authors’ contributions

TS contributed to the design of the study, collected, analyzed and interpreted the data and drafted the manuscript; RP participated in the coordination of the study and revised the manuscript critically for intellectual content. JL conceived the study, helped with statistical analyses and interpretation of the data and had general supervision of the study. All authors critically read and gave final approval of the version to be published.
